# P2X7 receptor as a key player in pathological pain: insights into Neuropathic, inflammatory, and cancer pain

**DOI:** 10.3389/fphar.2025.1585545

**Published:** 2025-07-11

**Authors:** Pan Jiang, Cai Wang, Fajing Jia, Hua Wu, Haihu Hao, Shaoze Jing

**Affiliations:** ^1^ Department of Orthopedics, Third Hospital of Shanxi Medical University, Shanxi Bethune Hospital, Shanxi Academy of Medical Sciences, Tongji Shanxi Hospital, Taiyuan, China; ^2^ Department of Cardiology, Shuozhou People’s Hospital, Shuozhou, China; ^3^ Department of Geriatric Medicine, Shanxi Bethune Hospital, Shanxi Academy of Medical Sciences, Third Hospital of Shanxi Medical University, Tongji Shanxi Hospital, Taiyuan, China

**Keywords:** P2X7 receptor, pathological pain, neuropathic pain, inflammatory pain, cancer pain

## Abstract

Purinergic P2X7 receptor (P2X7R) is a widely distributed, non-selective ATP-gated ion channel that plays a crucial role in the regulation of neuropathic pain, inflammatory pain, and cancer pain. Understanding the function and mechanisms of P2X7R in these various pain conditions, as well as utilizing P2X7R-targeted treatments, may offer a promising strategy for alleviating or resolving pathological pain. As a result, P2X7R and its antagonists have been the subject of extensive research, leading to the development of several P2X7R antagonists that have shown promising antinociceptive effects in numerous preclinical studies. However, further investigation and development are still necessary to fully realize their therapeutic potential. This review will provide an overview of the structure and function of P2X7R, its role in different pathological pain conditions, and the latest advances in the development of P2X7R antagonists, with the goal of offering new insights into the treatment of pathological pain.

## 1 Introduction

Pain is an unpleasant sensory and emotional experience, and it is one of the primary reasons patients seek medical care. Not only does pain affect the physical health of the individual, but chronic pain can also have a significant impact on mental health. Additionally, pain can impose a considerable economic burden on both the patient’s family and society at large. Pain varies widely in type and nature, with complex mechanisms underlying its onset. Investigating the generation and pathophysiology of pain provides essential insights for developing effective strategies for its relief or resolution in clinical settings, as well as for the discovery of novel analgesic therapies.

According to the International Association for the Study of Pain (IASP), pain is classified into three major types based on its underlying mechanisms: nociceptive pain, neuropathic pain, and nociplastic pain. Nociceptive pain is caused by the activation of nociceptors in response to actual or potential tissue damage (Costigan et al., 2009; Woolf, 2010; Abd-Elsayed and Deer, 2019). Neuropathic pain results from direct injury or dysfunction of the nervous system. Nociplastic pain, on the other hand, arises from central nervous system sensitization without clear evidence of nerve damage or tissue injury (Fitzcharles et al., 2021; Yoo and Kim, 2024). This review primarily focuses on neuropathic pain while also considering the broader category of pathological pain, which includes inflammatory pain and cancer pain.

Current research indicates that P2X7R, a type of extracellular adenosine triphosphate (ATP)-gated, non-selective cation channel (Surprenant et al., 1996; Hu et al., 2023; Zhang et al., 2024), plays a pivotal role in the initiation and maintenance of pathological pain through various peripheral and central mechanisms (Kim, 2016). Due to its significant regulatory role in the onset and progression of pathological pain, P2X7R has emerged as a promising therapeutic target for treating such conditions. As a result, it has become a focal point of research in the field of pain management in recent years.

## 2 The Biological characteristics of P2X7R

### 2.1 The structure of P2X7R

The human P2X7R is a transmembrane protein consisting of 595 amino acids (Roger et al., 2015; Li et al., 2020; Rotondo et al., 2022), located on chromosome 12 (Di Virgilio et al., 2017; Ren and Illes, 2022; Zhang et al., 2023). Both the N-terminus and C-terminus of P2X7R are intracellular. The N-terminus is relatively short, which has the function of regulating calcium influx (Boué-Grabot et al., 2000; Michel et al., 2008; Karasawa and Kawate, 2016) and are associated with interactions involving extracellular-regulated protein kinases. The C-terminus is longer compared to other P2X receptors and serves as the structural basis for its specialized functions. This region participates in many critical functions of P2X7R, such as modulating receptor activity, regulating signaling pathway activation, influencing cellular localization, facilitating protein-protein interactions, and supporting post-translational modifications (Gonnord et al., 2009; Costa-Junior et al., 2011; Lara et al., 2020). It also plays a crucial role in the transition from non-selective ion channel activity to the formation of a large pore upon receptor activation.

**FIGURE 1:**
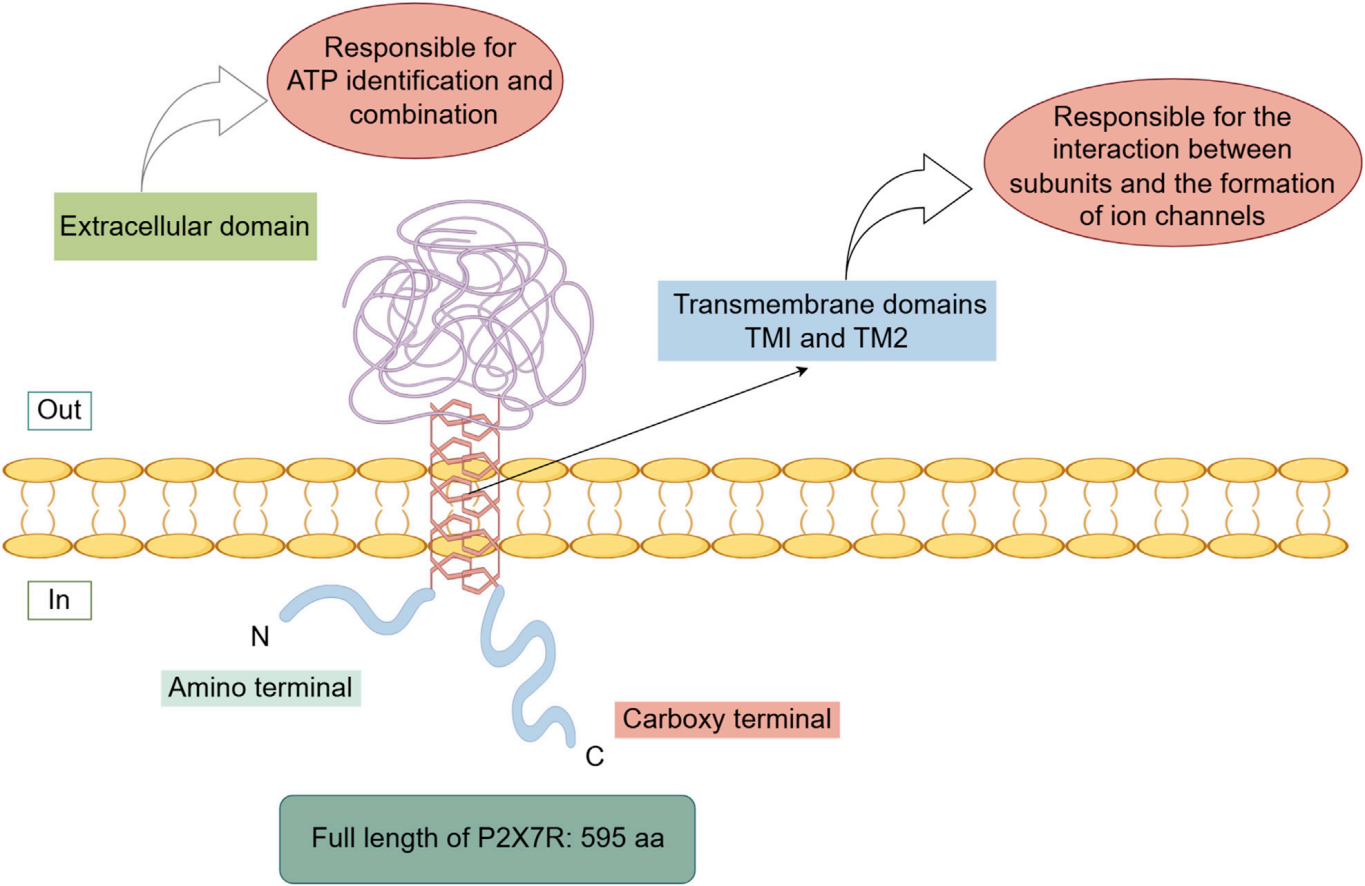
The structure of the P2X7R.

P2X7R primarily consists of two transmembrane domains (TM1 and TM2) and a large extracellular domain (Valera et al., 1994; Surprenant et al., 1996; Karasawa and Kawate, 2016; McCarthy et al., 2019). The transmembrane domains are composed of six α-helical structures, with three central α-helices (TM2) residing within the ion permeation pathway, and three peripheral α-helices (TM1) positioned outside this pathway. The extracellular domain is composed of 14 β-strands and four α-helices (Jiang et al., 2021). The transmembrane domains are primarily responsible for subunit interactions and ion channel formation, while the extracellular domain is predominantly involved in ATP recognition and binding (Figure 1).

### 2.2 The distribution of P2X7R

P2X7R is widely distributed throughout the human body, with expression observed in various cells within the nervous and immune systems. Additionally, it is found in numerous other cell types, including tumor cells, osteoblasts, osteoclasts, endothelial cells, epithelial cells, renal cells, and cardiomyocytes (Platania et al., 2019; Dong et al., 2020). Within the central nervous system, P2X7R is predominantly expressed in microglial cells but is also present in some neurons (Kan et al., 2019). In the peripheral nervous system, P2X7R is primarily localized in sensory neurons and certain non-neuronal cells (Wu et al., 2021). In the immune system, P2X7R is mainly found in macrophages, dendritic cells, B lymphocytes, T lymphocytes, and neutrophils, among other immune cells (von Mücke-Heim and Deussing, 2023). In tumor cells, P2X7R has been identified in various types of cancer, including breast cancer, prostate cancer, lung cancer, bone cancer, and gliomas (Zhang et al., 2020).

### 2.3 The function of P2X7R

P2X7R is classified as an ATP-activated, ligand-gated ion channel receptor. ATP serves as its natural agonist, and it needs higher concentration of ATP to activate P2X7 receptor compared with other P2XR subtypes. Upon brief ATP stimulation, P2X7R is activated, opening cation channels that lead to an influx of Na^+^ and Ca^2+^ and an efflux of K^+^, with the influx of Ca^2+^ being predominant (Surprenant et al., 1996; Di Virgilio et al., 2017; Adinolfi et al., 2018). Additionally, activated P2X7R facilitates the release of various inflammatory mediators, including interleukin-1β (IL-1β), interleukin-18 (IL-18), interleukin-6 (IL-6), and tumor necrosis factor α (TNF-α) (Perregaux and Gabel, 1998; Solle et al., 2001; Savio et al., 2018; Lara et al., 2020). Among these mediators, IL-1β induces the production of cyclooxygenase-2 (COX-2), nitric oxide synthase (NOS), and other enzymes, contributing to the development of hyperalgesia.

**FIGURE 2:**
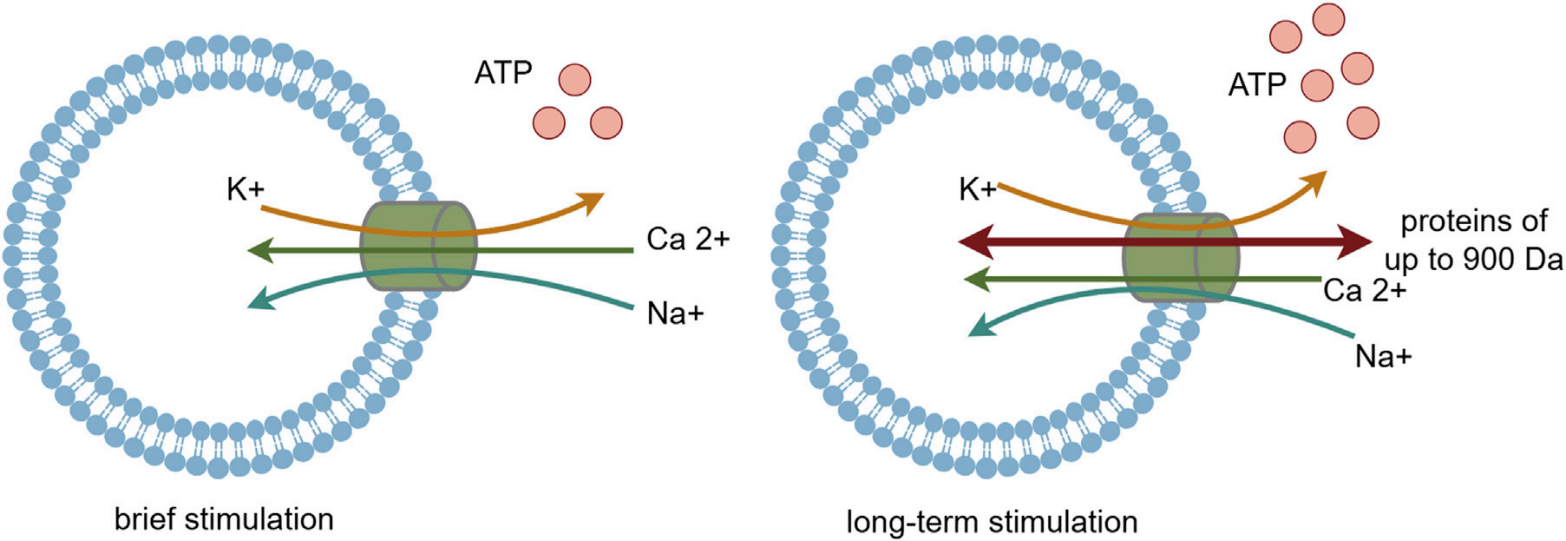
The two different states of P2X7R upon activation with extracellular ATP.

With prolonged ATP stimulation, P2X7R forms non-selective membrane pores that are permeable to large biomolecules up to 900 Da in molecular weight (Steinberg et al., 1987; Falzoni et al., 1995; Surprenant et al., 1996; Virginio et al., 1999; Shih et al., 2011; Hechler and Gachet, 2015). This allows these molecules to enter the cell, disrupting the balance of ion permeability across the cell membrane and ultimately leading to cell death (Figure 2). P2X7R plays a crucial role in various physiological and pathological processes, including inflammation, immune responses, cell death, and neurosignaling. Recent studies have also highlighted P2X7R’s pivotal role in neural conduction, particularly in the transmission of nociceptive signals (Drill et al., 2021), suggesting that P2X7R could be a potential target for the assessment and treatment of pathological pain.

## 3 P2X7R and pathological pain

### 3.1 P2X7R and neuropathic pain

Neuropathic pain arises from damage to the somatosensory nervous system and is associated with inflammatory stimuli that activate specific neuronal pathways involved in generating nociceptive responses (Finnerup et al., 2021). Research indicates that P2X7R plays a significant role in the development and persistence of neuropathic pain and ATP primarily activates purinergic receptors through damage-associated molecular patterns (DAMPs), subsequently inducing pain (Grace et al., 2018). Commonly used models for studying neuropathic pain include the chronic constriction injury (CCI) model, spared nerve injury (SNI) model, partial sciatic nerve ligation (PNL) model, spinal nerve ligation (SNL) model, and spinal cord transection (SCT) model. SNL, CCI, and SCT have all been shown to increase the expression of P2X7R in the spinal cord and dorsal root ganglia (DRG) (Yu et al., 2018b; Zhang et al., 2019). Studies by Chessell and Fulgenzi demonstrated that SNI and SNL do not induce hyperalgesia in P2X7R knockout mice (Chessell et al., 2005; Fulgenzi et al., 2008). Li et al. discovered that microvesicles (MVs) secreted by microglia in the cerebrospinal fluid of SNL rats rely on the P2X7R-p38 MAPK pathway to release IL-1β, which contributes to pain by inducing central sensitization. Furthermore, Li et al. found that in an SNL rat model, the expression and production of P2X7R in spinal microglia increase, leading to hyperalgesia (Li et al., 2017). Intrathecal injection of purinergic receptor inhibitors in this model resulted in a dose-dependent reduction in mechanical allodynia. Additionally, studies have shown that in a paclitaxel (a cytostatic/antitumor substance)-induced peripheral neuropathy model, the expression of P2X7R in spinal microglia is upregulated, which correlates with higher levels of mechanical allodynia (Ochi-ishi et al., 2014). Kataoka et al. (2009) demonstrated that in a rat diabetes model, exogenous ATP activated the nuclear factor of activated T cells (NFAT) through the P2X7R, which in turn promoted the release of chemokine (C-C motif) ligand 3 (CCL3) in microglia, ultimately leading to hyperalgesia in rats. Lu et al. (Lu et al., 2022) investigated a trigeminal neuralgia (TN) rat model and found that transplantation of olfactory ensheathing cells (OEC) to the site of infraorbital nerve ligation reduced P2X7R expression in the trigeminal ganglion. This intervention significantly increased the mechanical pain threshold in the rats’ faces, demonstrating a beneficial therapeutic effect for TN. Liu et al. examined the relationship between the pain-relieving effects of moxibustion (a traditional Chinese medicine practice that involves burning moxa, which is made from dried mugwort, near or on specific points of the body to promote healing) and P2X7R expression levels in the dorsal root ganglia (DRG) of rats subjected to chronic inflammatory stimulation through colorectal distension. They observed that moxibustion downregulated P2X7R expression, leading to a reduction in pain sensation (Liu et al., 2015b). In addition,In human studies, it has been observed that neuropathic pain patients exhibit significantly higher levels of P2X7R mRNA and protein in lymphocytes and monocytes compared to healthy controls (Luchting et al., 2016).

P2X7 receptors (P2X7Rs) are predominantly expressed in microglia, but they are also found in other glial cells, including astrocytes and oligodendrocytes. Activation of P2X7R triggers the release of pro-inflammatory cytokines and chemokines, reactive oxygen species (ROS), and excitotoxic transmitters such as glutamate and ATP. Additionally, P2X7R may play a role in signal transmission from glial cells to neurons under conditions of elevated ATP concentrations, particularly during neuropathic pain (Zhao et al., 2021).

These findings collectively suggest that P2X7R plays a crucial role in neuropathic pain, and targeting P2X7R may provide a potential therapeutic strategy to alleviate pain in such patients.

### 3.2 P2X7R and inflammatory pain

Inflammatory pain refers to pain that arises as a result of an inflammatory response triggered by peripheral tissue injury. Recent studies have shown that the P2X7R is closely associated with inflammatory responses and the development of pain, playing a crucial role in the progression of inflammatory pain. P2X7R is a well-characterized pro-inflammatory signaling receptor. During inflammation, elevated extracellular ATP levels activate P2X7R, leading to the release of various pro-inflammatory mediators, such as IL-1β, IL-18, TNF-α, and nitric oxide, which collectively contribute to the induction of inflammatory pain (Staunton et al., 2013; Allsopp et al., 2017). Furthermore, it has been reported that P2X7R activation promotes the assembly and recruitment of components of the NLRP3 inflammasome (Mariathasan et al., 2006; Karmakar et al., 2016). The NLRP3 inflammasome is an intracellular multiprotein complex consisting of the carrier protein caspase-1, the adaptor protein ASC, and the sensor protein NLRP3, which detects danger signals (Huang et al., 2021). Upon activation, NLRP3 triggers the conversion of full-length caspase-1 into its active form (He et al., 2016; Yang et al., 2019). Activated caspase-1 then cleaves pro-IL-1β, leading to the generation and release of mature IL-1β, thus initiating the inflammatory response (Perregaux and Gabel, 1994).

Research has shown that in a rat model of arthritis induced by monosodium iodoacetate (MIA), P2X7R expression is upregulated in spinal microglial cells, leading to the activation of PANX1 channels and the release of the pro-inflammatory cytokine IL-1β. Antagonizing or knocking out spinal microglial P2X7R alleviates MIA-induced mechanical allodynia (Mousseau et al., 2018). In a chronic pancreatitis visceral pain model in rats induced by 2% nitrobenzene sulfonic acid, P2X7R expression in the spinal cord is significantly upregulated. Furthermore, intrathecal injection of siRNA to knock down spinal P2X7R suppresses the expression of nociceptive behaviors in chronic pancreatitis rats (Liu et al., 2015a).

Additionally, Chessell et al. (2005) assessed the relative weight-bearing distribution between the FCA-injected (ipsilateral) and the contralateral hindpaws. Analysis of the ipsilateral/contralateral weight-bearing ration revealed a significant genotype effect. P2X7^−/−^ mice did not exhibit notable hypersensitivity at any time point after FCA injection, while P2X7^+/+^ mice displayed a significant reduction in the ipsilateral/contralateral ratio at both testing points. Similarly, Fulgenzi and colleagues found that in collagen-induced arthritis model mice and acetic acid-induced visceral pain model mice, the absence of the P2X7R gene prevents the manifestation of hyperalgesia (Fulgenzi et al., 2008). Numerous other studies support these findings. For instance, in carrageenan-induced arthritis, P2X7R mediates hyperalgesia through the release of pro-inflammatory factors and neutrophil migration. Teixeira et al. (2018) found that intra-articular injection of P2X7R agonist—2′(3′)-O-(4-Benzoylbenzoyl) adenosine 5-triphosphate (BzATP) into rat knees activates P2X7R, inducing the release of bradykinin, prostaglandins, sympathetic amines, and pro-inflammatory cytokines, which contribute to the maintenance of knee joint hyperalgesia in rats. In summary, P2X7R plays a crucial role in mediating the development of inflammatory pain.

### 3.3 P2X7R and cancer pain

Cancer pain refers to pain associated with cancer, caused directly or indirectly by the tumor. The pain is not limited to the tumor site and causes significant suffering for cancer patients. Cancer treatments often involve pain management therapies to alleviate this symptom. P2X7R has been shown to play a crucial role in cancer development, being highly expressed in various malignancies, including lung, colon (Rotondo et al., 2022), bone, prostate (Slater et al., 2004a), breast, and skin cancer (Greig et al., 2003; Slater et al., 2004b), as well as in neuroblastoma (Raffaghello et al., 2006), thyroid carcinoma (Solini et al., 2008), and B-cell chronic lymphocytic leukemia. It serves as a target for mediating cancer-related pain (Hansen et al., 2011; Yang et al., 2015; Bernier et al., 2018; Lara et al., 2020).

Research by Zhao et al. (2016) demonstrated that P2X7R plays a significant role in cancer pain. The researchers developed a bone cancer pain model by injecting Lewis lung cancer cells into the bone marrow cavity of the femur in both C57BL/6J wild-type mice and P2X7R knockout mice. Notably, they observed that the P2X7R knockout mice did not exhibit the pain behaviors typically induced by bone cancer in the model. Similarly, Yang et al. (2015) observed that in a rat model of bone cancer, spinal microglial P2X7R expression, phosphorylated p38 MAPK, and IL-18 levels were increased. Inhibition of the spinal P2X7R/p38/IL-18 pathway resulted in a reduction of pain behaviors in late-stage bone cancer. Similarly, Li et al. (2018) observed a mild increase in P2X7R expression in the ventrolateral periaqueductal gray (vlPAG) of the midbrain aqueduct in a rat model of bone cancer pain. Intraperitoneal injection of tramadol (10, 20, and 40 mg/kg) dose-dependently reduced pain-related behaviors in bone cancer pain rats, and further upregulated P2X7R expression in the vlPAG. The analgesic effect of tramadol was reversed by selective P2X7R antagonists. This suggests that tramadol may reduce norepinephrine (NE) reuptake in the vlPAG, thereby increasing local NE levels, which in turn elevates ATP concentrations and upregulates the expression of P2X7Rs in the vlPAG.

Research suggests that bone cancer pain shares characteristics with both inflammatory and neuropathic pain. Inhibiting P2X7R in spinal microglia can alleviate bone cancer pain by reducing spinal nerve hyperactivity through the p38/IL-18 pathway (Yang et al., 2015). Additionally, it can mitigate bone cancer pain by inhibiting NF-κB/p65, NLRP3-mediated inflammasome formation, and IL-1β expression (Wu et al., 2022). Moreover, P2X7R may not only be involved in mediating pain but also in tumor growth in cancers. Qiu et al., (2014) found that P2X7Rs are highly expressed in prostate cancer cells. Using siRNA technology to downregulate P2X7R expression significantly inhibited ATP or BzATP-induced migration and invasion of prostate cancer cells *in vitro*, as well as tumor invasion and metastasis in nude mice.

## 4 The application of P2X7R antagonists in pathological pain

Given the regulatory role of the P2X7R in pathological pain, this receptor has emerged as a promising target for the treatment of such pain and has garnered significant attention within the research community. To date, numerous P2X7R antagonists have been developed, and extensive preclinical studies have been conducted using rodent pain models (Table 1), including *in vitro* pharmacological investigations (Table 2). These studies have demonstrated that P2X7R antagonists effectively alleviate pain symptoms associated with neurological, inflammatory, and cancer-related conditions by inhibiting P2X7R activity.

**TABLE 1:** Summary of preclinical studies of P2X7 receptor antagonists in rodent pain models.

Pain model	Pain behavioural endpoints	P2X7R antagonist	Results	Mechanism of pain relief	Reference
A spinal nerve ligation model	Mechanical allodynia	A 740,003	Produce dose-dependent antinociception	Block agonist-evoked IL-1β release and pore formation	Honore et al. (2006)
The chronic constriction injury (CCI) model of the sciatic nerve	Tactile allodynia		Attenuate tactile allodynia		
A neuropathic model induced by vincristine	Tactile allodynia		Attenuate tactile allodynia		
Inflammatory pain model (carrageenan or CFA)	Thermal hyperalgesia		Significantly reduce thermal hyperalgesia		
The bone cancer pain rat model	The mechanical withdrawal threshold and thermal withdrawal latency values		The intra-vlPAG injection of A-740003 pretreatment partly but significantly antagonized the analgesic effect of tramadol on bone cancer pain rats	Block the release of IL-1β	Li et al. (2018)
The rat model with osteoarthritis (OA)	The test for the hindlimb weight-bearing asymmetry and paw withdrawal thresholds	AZD9056	Treatment with AZD9056 exerted pain-relieving and anti-inflammatory effects	Inhibit the activation of the MMP-13 and NF-κB pathways	Hu et al. (2016)
Bone cancer rat model	Hyperalgesia, mechanical pain	BBG	Significantly alleviate pain behavior in cancer rats	Reduce 5-HT level and Fos expression in the spinal cord	Huang et al. (2014)
Acute inflammatory pain model	Mechanoreceptive field, mechanical activation threshold and responses to noxious stimuli		The mustard oil (MO)-induced increase in nociceptive responses and decrease in activation threshold of medullary dorsal horn (MDH) nociceptive neurons	Attenuate the MO -induced MDH central sensitization	Itoh et al. (2011)
Postherpetic neuralgia (PHN) model	Mechanical paw withdrawal thresholds		PHN rats showed a lower paw withdrawal threshold	Inhibits endoplasmic reticulum stress and pyroptosis	Zhu et al. (2021)
Adult rat model with neonatal maternal deprivation (NMD)	Abdominal withdrawal reflex threshold to colorectal distension (CRD)	BBG	Microinjection of BBG into right insular cortex (IC) greatly increased CRD threshold in NMD rats	The enhanced activities of P2X7Rs in IC, likely through a presynaptic mechanism, contributed to visceral hypersensitivity of adult rats with NMD	Zhang et al. (2017)
		BzATP	Microinjection of BzATP into right IC significantly decreased CRD threshold in control rats		
Standard models of depression, mania and neuropathic pain	A combination of *in vitro* assays (calcium flux, radioligand binding, electrophysiology, IL-1β release)	JNJ-47965,567	Attenuate amphetamine-induced hyperactivity and exhibit modest, yet significant efficacy in the rat model of neuropathic pain. No efficacy was observed in forced swim test	Block the release of IL-1β	Bhattacharya et al. (2013)
The chronic constriction injury (CCI) model of the sciatic nerve	Mechanical withdrawal threshold (MWT) and the thermal withdrawal latency (TWL)	Gardenoside	Significantly improve the sciatica by partially restore the decrease of MWT and TWL in CCI rats	Regulate the P2X3 and the P2X7 expression on the ischiadic nerve	Yu et al. (2018a)
A rat model of neuropathic pain induced by chronic constriction injury (CCI)	The paw withdrawal latencies (PWLs) and the paw withdrawal mecha threshold (PWT)	Riluzole	The mechanical allodynia and thermal hyperalgesia in the hind limbs were significantly attenuated	Downregulate P2X7R expression and inhibit microglia activation in the spinal cord dorsal horn (SCDH)	Jiang et al. (2016)
Rat model of neuropathic pain	Mechanical allodynia and formalin induced pain behavior	A-438079	It was anti-allodynic and it attenuated formalin-induced nocifensive behaviors	Block the agonist-induced current in non-neuronal cells taken from the vicinity of dorsal root ganglion and reduce the amount of cytokine interleukin -1β released by peripheral macrophages in a dose-dependent manner	McGaraughty et al. (2007)
A rat model of cancer-induced bone pain	Early- and late-stage pain behavior	A839977	In awake animals, 40-mg/kg A839977 significantly reduced both early- and late-stage pain behavior. In contrast, no effect was observed in sham or vehicle-treated animals	Attenuate dorsal horn neuronal responses in a modality and intensity-specific way	Falk et al. (2015)
A model for aqueous deficient Dry eye disease (DED)	Orbicularis oculi muscle activity (OOemg)	A804598	Local microinjection in the caudal trigeminal brainstem of A804598 greatly reduced hypertonic saline (HS)-evoked OOemg activity in all DED groups, while responses in sham groups were not affected	Activation of P2X7R at central and peripheral sites in trigeminal pain pathways contributed to an increase in ocular hyperalgesia and microglia activation in DED males and females	Bereiter et al. (2022)

**TABLE 2:** P2X7 antagonists and *in vitro* pharmacology.

Antagonist	*In vitro* model system pKi/IC_50_/EC_50_	Biological activity	Reference
A-740003	hP2X7 IC_50_ = 40 nM rP2X7 IC_50_ = 18 nM	Effective and highly selective	Honore et al. (2006)
	IC_50_ = 156 nM (release of IL-1β in human THP-1 cells)	Effectively inhibit the release of IL-1β	
	IC_50_ = 92 nM (formation of membrane pores in human THP-1 cells)	Effectively inhibit the formation of membrane pores	
	IC_50_ > 10 muM (at other P2 receptors and an array of other neurotransmitter and peptide receptors, ion channels, reuptake sites, and enzymes)	Weak or no activity	
	rP2X7 pIC_50_ = 7.0–7.3	Potently inhibit Ca^2+^ flux, Yo-Pro uptake, and IL-1β release	Carroll et al. (2009)
A-847227	hP2X7 pIC_50_ = 8.3	Effectively inhibit the release of IL-1β	Carroll et al. (2009)
JNJ-47965,567	human blood pKi = 6.7 ± 0.07; human monocytes pKi = 7.5 ± 0.07; rat microglia pKi = 7.1 ± 0.1	Centrally permeable, high-affinity, selective	Bhattacharya et al. (2013)
SB203580	hP2X7 IC_50_ = 5 μM	Low-affinity, non-competitive	Caseley et al. (2015)
KN62	hP2X7 IC_50_: ∼100 nM	Potent and selective human P2X7 antagonist	Caseley et al. (2015)
	rmP2X7 IC_50_ = 86 ± 19 (nM) hP2X7 IC_50_ = 130 ± 40 (nM)	A potent hP2X7 receptor antagonist, and there is no significant difference compared to rmP2X7 receptor	Bradley et al. (2011)
	hP2X7 pIC_50_ ∼ 6.7 rP2X7 pIC_50_ < 4	Having potent activity at the human receptor but lacking activity at the rat receptor	Donnelly-Roberts and Jarvis (2007)
AZ116453743	hP2X7 IC_50_: ∼90 nM rP2X7 pIC_50_: over 500-fold less effective	A highly selective and potent antagonist at human but not rat P2X 7 receptors	Caseley et al. (2015)
	rmP2X7 IC_50_ = 23 ± 3 (nM); hP2X7 IC_50_ = 31 ± 3 (nM)	A potent hP2X7 receptor antagonist, and there is no significant difference compared to rmP2X7 receptor	Bradley et al. (2011)
	rP2X7 pIC_50 _< 5 hP2X7 pIC_50_ ∼ 8	Shows a significant reduction in potency at the rat receptor relative to human	Stokes et al. (2006)
BzATP	rmP2X7 EC_50_ = 58 ± 4 µM hP2X7 EC_50_ = 30 ± 2 µM	Full agonist in activating the rmP2X7 receptor	Bradley et al. (2011)
A-438079	rmP2X7 IC_50_ = 297 ± 24 nM hP2X7 IC_50_ = 493 ± 94 nM	Effective in inhibiting the hP2X7 receptor as well as the rodent P2X7 receptors	Bradley et al. (2011)
GSK314181A	rP2X7 pIC_50_ = 9.0	To be potent at the rat P2X7 receptor	Carroll et al. (2009)
Cycloalkyl	hP2X7 pIC_50_ = 7.7	Showing potent activity	
Aryl spirocyclohexyl	hP2X7 pIC_50_ = 7.3	Showing potent activity	

Notes: rmP2X7: rhesus macaque P2X7; rP2X7: rat P2X7; hP2X7: human P2X7.

### 4.1 The application of P2X7R antagonists in neuropathic pain

P2X7R antagonists have been shown to alleviate neuropathic pain while also enhancing immune suppression and neuroprotection, a finding confirmed relatively early in various preclinical studies (Honore et al., 2006; Carroll et al., 2009; Andó et al., 2010; Kobayashi et al., 2011). Studies indicate that in a rat spinal cord injury (SCI) model, administration of the P2X7R antagonist Brilliant Blue G (BBG) significantly inhibits neuronal apoptosis. Similarly, intrathecal injection of the P2X7R antagonist A740003 reduces mechanical allodynia induced by SNL and CCI (Chessell et al., 2005). Moreover, intrathecal injection of P2X7R antagonist A438079 alleviates hyperalgesia in mice following trigeminal nerve transection. Bhattacharya et al. (2013) demonstrated that using the P2X7R antagonist JNJ-47965567 with central permeability, high affinity and selectivity in rodent models of central nervous system neuropathic pain reduces Bz-ATP-induced IL-1β release, thereby reducing pain. Yu et al. (Jiang et al., 2016; Yu et al., 2018a) found that non-selective P2X7R inhibitors, such as geniposide and riluzole, inhibit the release of immune factors like NOS, IL-1β, and TNF-α, while also suppressing activation of the MAPK signaling pathway. This ultimately reduces the activation of central microglia and alleviates neuropathic pain in rats. Furthermore, in diabetic neuropathic pain, the P2X7R antagonist BBG can suppress P2X7R-mediated hippocampal glial cell excitation and IL-1β production and release, thereby alleviating diabetes-induced neuropathic pain. In models of sciatic nerve injury-induced neuropathic pain, the application of P2X7R antagonists BBG and A740003 significantly reduces sciatic pain (Li et al., 2018). Additionally, P2X7R antagonist A740003 alleviates CCI and vincristine-induced neuropathic pain, while A438079 attenuates neuropathic pain induced by SNL and CCI (McGaraughty et al., 2007). Thus, the use of P2X7R antagonists has demonstrated efficacy in treating neuropathic pain.

### 4.2 The application of P2X7R antagonists in inflammatory pain

Given the relationship between P2X7R and inflammatory pain, numerous P2X7R antagonists have been developed to alleviate inflammatory pain, and their efficacy has been confirmed in extensive studies. Research has shown that intra-articular injection of Freund’s Complete Adjuvant (FCA) significantly lowers the nociceptive threshold in rats, increasing P2X7R expression at the mRNA level in nerve fibers and blood vessels of the hind paws. The use of the P2X7R antagonist A740003 markedly reduces inflammatory pain in FCA-induced arthritis model rats and decreases P2X7R expression at the inflammation site. Clark et al. (2010) suggested that P2X7R contributes to pain by targeting microglial cells to release IL-1β, and the administration of P2X7R antagonists, such as A740003, A438079, and oxATP, can inhibit IL-1β release. Zhang et al. (2017) discovered that P2X7R sensitizes rat Insular cortex (IC) neurons through a presynaptic mechanism, and BBG can block P2X7R at presynaptic sites, alleviating inflammatory pain. Teixeira et al. (2018) found that the P2X7R antagonist A740003 could block bradykinin- and dopamine-induced joint hyperalgesia. In animal models of knee osteoarthritis, the use of P2X7R antagonists significantly reduced the concentration of inflammatory factors such as IL-1β, IL-6, TNF-α, matrix metalloproteinase (MMP-13), substance P, and prostaglandin E2 (PGE2) in the joints, thereby alleviating osteoarthritic pain (Hu et al., 2016). Additionally, Itoh et al. (2011) found that the P2X7R antagonist BBG effectively alleviates inflammatory pain induced by mustard oil. A438079 relieves pain behaviors induced by FCA, bee venom, and carrageenan, while A740003 and A438079 reduce spontaneous nociceptive behaviors in the second phase of the formalin pain model, which corresponds to the peripheral inflammatory pain stage. Other studies have shown that the P2X7R antagonist oxATP effectively inhibits inflammatory pain induced by FCA, carrageenan, collagen, lipopolysaccharide, and mustard oil (Fulgenzi et al., 2005; Fulgenzi et al., 2008; Clark et al., 2010). These findings indicate that widely used P2X7R antagonists exhibit significant analgesic effects across various types of inflammatory pain.

### 4.3 The application of P2X7R antagonists in cancer pain

In addition to their applications in neuropathic and inflammatory pain, P2X7R antagonists are increasingly being utilized for the relief of cancer pain. These include polyene sulfonate acid dyes such as BBG, natural products like emodin and colchicine, and newly synthesized molecules such as A438079, A740003, A804598, AZ 10606120, AZ 11645373, KN-62, and A839977 (Marques-da-Silva et al., 2011; Faria et al., 2012; Jelassi et al., 2013; Custer et al., 2018; Wu et al., 2023). BBG can reduce inflammation and alleviate cancer pain by inhibiting glial cell activation and blocking the release of IL-1β and IL-18 (Halvorson et al., 2005; Yue et al., 2017). Emodin alleviates cancer pain by inhibiting P2X7R activation, reducing ATP-induced intracellular Ca^2+^ concentrations, decreasing IL-1β release, and lowering ROS production (Jelassi et al., 2013). Colchicine mitigates cancer pain by reducing ROS and NO production, as well as IL-1β secretion. Compounds like A438079, A804598, and AZ 1164537 relieve cancer pain by inhibiting microglial function and inflammatory reactions (Jiang et al., 2017; Rosli et al., 2019; Liu et al., 2020), whereas A740003 and AZ 10606120 alleviate cancer pain by reducing calcium influx (Allsopp et al., 2017; Li et al., 2021). KN-62 not only reduces ATP-dependent Ca^2+^ influx and ethidium bromide uptake but also decreases IL-1β secretion to relieve cancer pain. Falk et al. (2015) demonstrated that intrathecal injection of the P2X7R-specific inhibitor A839977 significantly reduced pain in both the initial and maintenance phases of the breast cancer pain (BCP) model in rats injected with MRMT-1 breast cancer cells. Huang et al. (2014) found that BBG effectively relieved pain in a rat model of bone cancer. Additionally, Falk et al. (2015) discovered that A839977 could effectively alleviate early and late-stage pain behaviors in animal models of bone cancer pain. Honore et al. (2006) found that A740003 had a significant analgesic effect on rats with bone cancer pain.

## 5 Conclusion

P2X7 is a purinergic receptor involved in pathological pain, exhibiting high expression levels in neuropathic pain, inflammatory pain, and cancer pain. By regulating the opening of cation channels, promoting the release of various inflammatory mediators, and modulating the transmission of nociceptive signals, P2X7R mediates the generation of pain. Therapeutic strategies targeting P2X7R are being extensively researched in the context of pathological pain. In recent years, numerous P2X7R antagonists have been developed, all showing promising pharmacological properties, laying the groundwork for exploring P2X7R’s role in pain management. It has been confirmed that P2X7R plays a crucial role in the initiation and progression of inflammation as well as in the process of pain, with antagonism of P2X7R alleviating pain. However, despite initial insights into the role of P2X7R in pathological pain, many questions remain unresolved, such as the need for further exploration of the mechanisms of action of P2X7R and comprehensive studies on antagonists. Therefore, deeper investigation into the detailed mechanisms of P2X7R in pathological pain, including its effects in different types of diseases and pain conditions, is required. Elucidating its function will provide guidance for the discovery of analgesic drugs and innovations in pain relief methods. Additionally, more clinical trials are needed to confirm the efficacy of P2X7R antagonists—efforts which we must collectively undertake.

## Data Availability

The original contributions presented in the study are included in the article/supplementary material, further inquiries can be directed to the corresponding authors.

## Abbreviations

NOS, Nitric oxide synthase; DAMPs, Damage-associated molecular patterns; CCI, Chronic constriction injury; SNI, Spared nerve injury; PNL, Partial sciatic nerve ligation; SNL, Spinal nerve ligation; SCT, Spinal cord transection; DRG, Dorsal root ganglia; MVs, Microvesicles; MAPK, Mitogen-activated protein kinase; MIA, Monosodium iodoacetate; PANX1, Pannexin1; CFA, Complete Freund’s adjuvant; BzATP, Ammonium salt; vlPAG, Ventrolateral periaqueductal gray; NF-κB, Nuclear factor kappa-B; NLRP3, Nucleotide-oligomerization domain (NOD)-like receptor pyrin domain containing protein 3; SCI, Spinal cord injury; BBG, Brilliant blue g; MMP-13, Matrix metallopeptidase 13; PGE2, Prostaglandin E2; BCP, Breast cancer pain; MRMT-1, Mammary rat metastasis tumor.
